# Are hydraulic patterns of lianas different from trees? New insights from *Hedera helix*

**DOI:** 10.1093/jxb/erz071

**Published:** 2019-02-22

**Authors:** Andrea Ganthaler, Katharina Marx, Barbara Beikircher, Stefan Mayr

**Affiliations:** Department of Botany, University of Innsbruck, Sternwartestrasse, Innsbruck, Austria

**Keywords:** Climber, drought resistance, embolism, leaf dimorphism, osmotic adaptation, plant water relations, xylem anatomy

## Abstract

Lianas form long and flexible but disproportionately narrow stems, and thus require particular strategies to maintain the integrity of xylem water transport and ensure supply to large crown areas. The hydraulic architecture of lianas and the respective within-plant coordination of transport efficiency and safety, and the underlying anatomical variations in xylem, are largely unexplored. We analysed *Hedera helix*, a liana widespread in European temperate forests, with respect to hydraulic and xylem anatomical variations between the main stem and branches, between juvenile and adult life phases, and along the vertical axis. Main stems were significantly less embolism resistant but exhibited a higher hydraulic conductivity than branches. In branches, the cell turgor loss point of leaves decreased, while the embolism resistance and conductivity of xylem, as well as conduit diameters, increased with height. High water-transport capacities allow ivy to compensate for the small cross-section of stems, while the limited resistance to drought-induced xylem dysfunction of the main stem is probably linked to conservative stomatal regulation. Pronounced differences in xylem anatomy, hydraulic efficiency, and safety between the main stem and branches and along the vertical axis are surprisingly similar to those of self-supporting plants, and indicate that the coordination of carbon and water economies requires similar internal adjustments in tall plants.

## Teaser

Common ivy ensures water supply of the crown by well-balanced within-plant adjustments of hydraulic efficiency and safety. Patterns correspond to variations in xylem anatomy and are similar to those of self-supporting trees.

## Introduction

Lianas and other climbing plants are characterized by large crown areas supported by long and flexible but disproportionately narrow main stems (Schnitzer and Bongers, 2002; Wyka *et al.*, 2013; Rosell and Olson, 2014). The discrepancy between leaf area and supporting stem cross-sectional area has consequences for both static and hydraulic aspects (Ewers, 1985). On the one hand, the mechanical function of the stem, and thus resource allocation to the xylem, can be reduced, as climbers are not self-supporting but rely on neighbouring trees or other structures to support their weight. Water transport and regulation, on the other hand, have to be optimized with regard to capacity and safety by a hydraulic architecture adapted to this growth form (Ewers and Fisher, 1991).

Lianas apparently show strong selection for conductive efficiency and exhibit higher specific conductivities and higher sap flux compared with angiosperm and gymnosperm trees (Ewers, 1985; Chen *et al.*, 2017; Ichihashi *et al.*, 2017). Comparisons of western poison oak plants growing as vines or as shrubs (Gartner, 1991), and of *Bauhinia* species with different growth forms (Ewers and Fisher, 1991), indicate that individuals with narrow vine stems are able to achieve the same leaf specific conductivity as individuals growing as shrubs with wider stems. Higher hydraulic efficiency is thought to be based on the formation of wider vessels in the xylem. Extremely wide vessels can be found in lianas from tropical rainforests (Gartner *et al.*, 1990; Ewers and Fisher, 1991), but also in woody climbers from temperate regions (Tibbetts and Ewers, 2000). However, Rosell and Olson (2014) pointed out that climbing plants exhibit a higher variance in vessel diameter, rather than a higher mean vessel diameter, and Olson *et al.* (2014) showed that, for a given stem length, non-self-supporting plants have only slightly higher mean hydraulic vessel diameters than self-supporting plants. Tall plants need to optimize biomass allocation and xylem investment to guarantee efficient and safe long-distance water transport (Petit *et al.*, 2016). Hydraulic optimality models suggest that vessel diameters therefore increase from stem tips toward the stem base following a power law (Anfodillo *et al.*, 2006; Olson *et al.*, 2014). This axial conduit widening can substantially buffer the effect of increasing path length on the cumulative hydrodynamic resistance (West *et al.*, 1999; Mencuccini *et al.*, 2007).

In contrast to their increased hydraulic efficiency, lianas were found to be less tolerant to drought-induced cavitation than co-occurring trees (Van der Sande *et al.*, 2013; Chen *et al.*, 2017). However, the combination of wide and efficient vessels together with embolism-resistant narrow vessels and tracheids provides a small but sufficient transport capacity during dry periods. This pronounced vessel dimorphism is highly characteristic of lianas and can be interpreted as a strategy to combine high hydraulic efficiency with sufficient hydraulic safety (Carlquist, 1985). Trees, in contrast, rarely achieve both high efficiency and high safety (Gleason *et al.*, 2016). Moreover, several hypotheses for the abundant occurrence of starch-rich parenchyma in woody climbers exist, especially with respect to producing positive pressure and providing water and osmotic agents to enable repair mechanisms (Cochard *et al.*, 1994; Tibbetts and Ewers, 2000; Chen *et al.*, 2017). Many lianas also show a very sensitive stomatal response to increasing vapour pressure deficits during the day (Leuzinger *et al.*, 2011; Chen *et al.*, 2017).

Woody climbers are a widespread growth form in tropical rainforests, but also influence the dynamics and function of temperate forests by affecting species composition, tree regeneration, and water dynamics (Schnitzer and Bongers, 2002; Castagneri *et al.*, 2013; Ichihashi *et al.*, 2017). One of the most widespread lianas in temperate European forests is common ivy (*Hedera helix* L., Araliaceae). Common ivy climbs with anchoring roots on the host tree, supported by stable, upright elements, forms foliated branches from the base to the top, and can reach a height of up to 30 m (Metcalfe, 2005). The species is tolerant to a wide variety of substrates, but grows most vigorously in shaded, moist sites. It is limited in its range by low winter temperatures (Andergassen and Bauer, 2002) and may profit in the future from rising temperatures (Walther, 2002; Heuzé *et al.*, 2009; Rehm *et al.*, 2014) and elevated CO_2_ concentrations (Zotz *et al.*, 2006). Ivy is a classical example of a heterophyllous plant, forming a juvenile and an adult phenotype depending on the plant height and light conditions. For instance, ivy plants growing on sun-exposed sites produce juvenile shoots up to a height of ~1.2 m and adult shoots above this height. The juvenile stage has vertically flattened branches with three- or five-lobed leaves and faster shoot growth but fewer leaves; in the adult stage, shorter, flowering shoots with numerous spirally arranged entire leaves are generated (Miller and Goodin, 1976; Metcalfe, 2005). The two phenotypes also differ in their photosynthetic capacity, stomatal and residual conductance leaf thickness, and number of chloroplasts (Bauer and Bauer, 1980; Hoflacher and Bauer, 1982), as well as frost resistance and timing of frost hardening (Andergassen and Bauer, 2002; Rehm *et al.*, 2014).

Studies on *H. helix* are interesting from a physiological point of view for a variety of reasons. First, it is a widespread representative of lianas and thus offers general insights into structure–function relationships of non-self-supporting plants. Being a temperate liana, it can also be compared with numerous comparably well-studied temperate host trees. Second, ivy is a prominent example of heterophylly by forming two different phenotypes with leaves adapted to low- and high-light conditions. Third, this structural parasite is known to affect temperate forests, as it intensively interacts and competes with host trees for light and water. The hydraulic architecture of ivy and other temperate lianas has received little attention. The only available study on *H. helix* (Leuzinger *et al.* 2011) examined water relations, and reported surprisingly constant leaf water potential and sap flow across different environmental conditions, coupled with sensitive stomatal regulation. The authors assumed that the conservative flow control may have evolved as an adaptation to the potentially high vulnerability of the xylem to drought-induced embolism, and that potentially low transport resistances ensure moderate water potentials under transpiration, for example, at low vapour pressure deficits; however, this has not yet been confirmed.

In the present study, we analysed water transport efficiency and safety, and the underlying xylem anatomy and leaf cell hydraulic parameters, in *H. helix*. We expected this liana to exhibit overall high hydraulic efficiency but low hydraulic safety. Furthermore, we hypothesized that within-plant hydraulic patterns are affected by the demands of the climbing habit and the reduced mechanical function of the xylem, and thus differ from the hydraulic architecture of self-supporting trees.

## Materials and methods

### Study site and plant material

The study was conducted in the Botanical Garden of the University of Innsbruck, Austria (47.267°N, 11.393°E; 574 m above sea level), with an average annual precipitation of 896.5 mm and an average air temperature of 8.5 °C in the 30-year mean (ZAMG, 2002). Ivy plants with a growth height of about 15 m and stem diameters up to 9 cm were selected; these plants were climbing on an oak (*Quercus robur*), a pine (*Pinus sylvestris*), and the institute building. Branches (0.7–1 m long) were sampled by the use of telescopic scissors and the help of tree climbers at heights of 0–1 m (juvenile branches) and 1–2 m, 7–8 m, and 12–13 m (adult branches). Samples were immediately re-cut several times under water and rehydrated for at least 12 hours (Beikircher and Mayr, 2016). After branch sampling, stem sections were sampled in a similar fashion with a motor saw at 1–2 m and 7–8 m height from the main stems and then re-cut under water with a sharp carving knife. All measurements were made during the summer months between April and November.

### Pressure–volume analysis

Leaf hydraulic parameters, including osmotic potential at saturation (Ψ_o_), turgor loss point (Ψ_tlp_), and modulus of elasticity (ε), were analysed via pressure–volume analyses (Tyree and Hammel, 1972) on five fully developed leaves of different plants, from each sampling height. Leaves were saturated before dehydration was applied and repeated measurements of Ψ and weight were made. Ψ was measured with a pressure chamber (model 1000 ‘upgraded to 100 bar’ pressure chamber; PMS Instrument, Albany, OR, USA). The relative water content was calculated from saturated, fresh, and dry weights and plotted against the inverse leaf Ψ (1/Ψ). The turgescent section was fitted with a parabolic function and the osmotic section with a linear regression according to Boyle’s law using Fig.P 2006 (Fig.P Software Inc., Hamilton, ON, Canada) to determine Ψ_tlp_ (the intercept of linear and parabolic function), Ψ_o_ (the negative inverse of the linear graph portion’s intercept with the y-axis), and ε (the slope of the curve above the turgor loss point; Ganthaler and Mayr, 2015). Parameters were calculated individually per leaf and then averaged per sampling height.

### Hydraulic conductivity

Branch sections (10 per sampling height, up to 12 cm long and 6 mm in diameter) were excised under water, at least 10 cm distant from the basal branch end. Sample ends were re-cut several times by ~5 mm with a sharp wood-carving knife, decorticated, and sealed in the hydraulic measuring system. Stem sections (five per sampling height, up to 35 cm long and 40 mm in diameter) were treated likewise with a carving knife and connected to the measuring system with a rubber adaptor.

The flow rate was determined with a flow meter (mini Cori-Flow 100 g h^−1^, Bronkhorst High Tech, Ruurlo, The Netherlands) at a pressure of 5 kPa using distilled, filtered (0.22 µm), and degassed water containing 0.005% (v/v) Micropur (Katadyn Products, Kemptthal, Switzerland) to prevent microbial growth. Samples were repeatedly flushed at 70 kPa for 15 min to ensure that native embolism was removed and measurements represented maximum conductance. The specific hydraulic conductivity (*k*_s_; m^2^ s^−1^ Pa^−1^) was calculated as

$$\begin{array}{l} k_{s}=Q\times l÷(A_{c}\times \Delta P) \end{array}$$

where Q is the volume flow rate (expressed in m^3^ s^−1^), l is the length of the sample (in m), *A*_c_ is the xylem cross-sectional area (in m^2^), and ΔP is the pressure difference between the segment ends (in Pa). Calculations were corrected to 20 °C to account for changes in fluid viscosity with temperature.

### Vulnerability analysis

Vulnerability curves were analysed on five to nine samples of stems and branches from each height by using the cavitron technique (Cochard *et al.*, 2005). This method uses centrifugal force to increase the water tension in a xylem segment while simultaneously measuring the loss of conductance. Fully hydrated branch and stem segments of 275 mm length were fixed in a custom-built 280 mm rotor in the centrifuge (Sorvall RC-5 Superspeed Refrigerated Centrifuge, Thermo Fisher Scientific, Waltham, MA, USA). Sample ends were positioned in upstream and downstream reservoirs, which were filled with distilled, filtered (0.22 μm), and degassed water containing 0.005% (v/v) Micropur. The temperature was set to 10 °C. Before the first measurement of hydraulic conductance, samples were equilibrated for 20 min at low rotating speed (inducing –0.25 MPa). Then, the rotational speed was increased stepwise to the next target pressure and hydraulic conductance was measured after an equilibration time of 1 min. The moving water meniscus in the upstream cuvette was observed using a high-resolution camera (Motic MC 2000, Motic China Group Co., Ltd) and the flow rate and hydraulic conductance were calculated. Percentage loss of conductivity (PLC) was calculated from the ratio of actual (after inducing a given Ψ) to the maximum (first measurement at −0.25 MPa) hydraulic conductance (Beikircher *et al.*, 2010).

Curves of each sample were fitted with an exponential sigmoidal equation according to Pammenter and Vander Willigen (1998) as

$$\begin{array}{l} PLC=100÷\left(1+exp\left(a\left(\Psi -\Psi_{LC50}\right)\right)\right) \end{array}$$

where *a* is a constant related to the curve slope and Ψ_LC50_ is the xylem Ψ at 50% loss of conductivity. Fitting of the curves was performed with Fig.P 2006 (Fig.P Software Inc., Hamilton, ON, Canada). We also calculated Ψ at 12% and 88% PLC (Ψ_LC12_, Ψ_LC88_) and averaged the results per sampling height.

Segments of the main stem had to be split lengthwise to fit into the cavitron rotor. For this, sticks of 2–3 cm diameter and 30 cm length were axially split from the main stem. This was performed under water with a carving knife, following the fibre structure of the wood and thus minimizing the number of open vessels on the lateral sites. We thus expected potential shifts in the vulnerability curve to be minor (open vessels would be emptied and thus not included in the analysis, potentially causing an underestimation of the vulnerability). An artefact due to the sample length can be excluded as the maximum conduit length was 73.2±3.3 mm in adult branches, 79.8±2.7 mm in juvenile branches, and 109.8±3.8 mm in the main stem (analysed by forcing compressed air through five branch and stem segments, respectively; Ewers and Fisher, 1989; Beikircher and Mayr, 2016). Vessels were thus distinctly shorter than the rotor diameter (280 mm) and could not be opened at both ends by sample preparation. Accordingly, the sigmoidal shape of the vulnerability curves from the stem and branches of *H. helix* also indicated that open vessel artefacts were not relevant.

### Wood anatomical measurements

Branch and stem sections previously used for hydraulic conductivity measurements were soaked in ethanol/glycerol/water solution (1:1:1, v/v/v) for at least 2 weeks. Cross-sections (8 µm thickness) were cut with a microtome (Schlittenmikrotom G.S.L. 1, Schenkung Dapples, Zürich, Switzerland), stained with Etzold solution (fuchsin/chrysoidin/Astra blue), and analysed with a light microscope (Olympus BX41; Olympus Austria, Wien, Austria) interfaced with a digital camera (ProgRes CT3, Jenoptik, Jena, Germany). In randomly selected radial sectors, areas of all conduits (68–174 per sample) within the outermost two growth rings were analysed with the image analysis software ImageJ 1.45 (National Institutes of Health, MD, USA). The diameters were calculated from conduit areas, assuming a circular shape, averaged per sample, and the mean diameter (*d*_mean_) per species was calculated from these values. The average hydraulic conduit diameter (*d*_h_) was calculated from the diameter of all analysed conduits Kolb and Sperry (1999) as

$$\begin{array}{l} d_{h}=\Sigma d^{5}÷\Sigma d^{4} \end{array}$$

To characterize conduit wall reinforcement, the wall thickness to span ratio (*t*/*b*)^2^ (Hacke and Sperry, 2001) was assessed. The thickness of tangential interconduit walls (*t*) and the conduit diameter (*b*) were measured for conduit pairs with average diameters within *d*_h_ ±1 µm (five conduit pairs per sample). The values were averaged per sample, and the mean (*t*/*b*)^2^ per sampling point was calculated from these values. In addition, the percentage of xylem area covered by conduit lumen (area_lumen_) and the percentage of the cross-sectional area occupied by the central pith parenchyma (area_pith_) was measured.

### Annual growth

On the main stem of three individual ivy plants, discs were taken at 1.5 m and 7.5 m above ground for determination of age and measurement of annual ring width. Measurements were made of each disc and results were averaged per sampling height.

### Statistical analysis

**Fig. 1.**
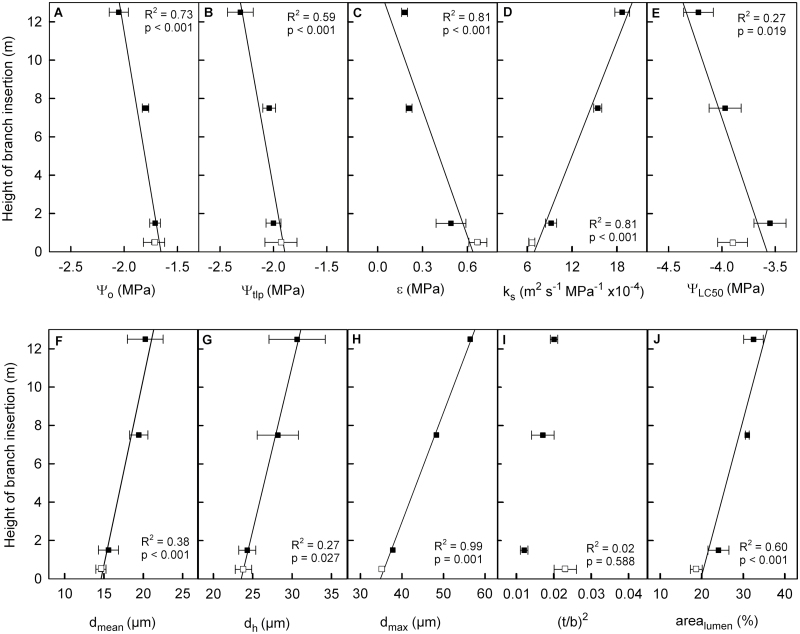
Correlation of hydraulic and anatomical parameters with sampling height. (A) Leaf osmotic potential at full turgor (Ψ_o_), (B) cell turgor loss point (Ψ_tlp_), (C) modulus of elasticity (ε), (D) specific hydraulic conductivity (*k*_s_), (E) water potential at 50% loss of conductivity (Ψ_LC50_), (F) mean conduit diameter (*d*_mean_), (G) mean hydraulic diameter (*d*_h_), (H) maximum conduit diameter (*d*_max_), (I) cell wall thickness to span ratio [(*t*/*b*)^2^], and (J) percentage of xylem area covered by conduit lumen (area_lumen_) of juvenile branches (empty squares) and adult branches (filled squares). Data presented are means ±SE.

Differences were tested with Student’s *t*-test after testing for homogeneity of variance (Levene test) and for Gaussian distribution (Kolmogorov–Smirnov test). Correlation analyses were carried out using Pearson product-moment correlation and were based on all individual measurement values (Fig. 1) or the mean values of branches and main stems from different sampling heights (Fig. 4). In the second case, the standard error was included in statistical tests as a weighting factor. All tests (two-tailed) were performed pairwise at a probability level of 5% using SPSS version 24 (IBM Corporation, Armonk, NY, USA). All values are reported as mean ±SE.

## Results

### Pressure–volume analysis

At the lowest sampling heights, Ψ_o_ of leaves was least negative (approximately –1.7 MPa) and similar in juvenile and adult leaves. It decreased with height to –2.05 MPa at 12–13 m (Table 1). Ψ_tlp_ followed a similar trend and decreased from –1.93 MPa in juvenile leaves to –2.31 MPa in adult leaves from the highest sampling point. Juvenile leaves had a higher ε than adult leaves, indicating less elastic cells in the juvenile foliage. Moreover, ε was significantly lower in adult leaves from the top than from the base of the plant. All three leaf hydraulic parameters correlated with sampling height (Fig. 1A–C), whether only adult branches or all branches were included in the analysis.

**Table 1.** Pressure–volume analysis of *Hedera helix*

	Juvenile leaves	Adult leaves		
Parameter	*0–1 m*	*1–2 m*	*7–8 m*	*12–13 m*
ψ_o_ (MPa)	–1.72±0.10^a^	–1.71±0.05^a^	–1.80±0.03^a^	–2.05±0.09^b^
ψ_tlp_ (MPa)	–1.93±0.15^a^	–2.00±0.07^a^	–2.04±0.06^a^	–2.31±0.12^b^
ε (MPa)	0.67±0.06^a^	0.49±0.10^b^	0.21±0.02^c^	0.18±0.02^c^

Osmotic potential at saturation (Ψ_o_), turgor loss point (Ψ_tlp_), and modulus of elasticity (ε) of juvenile leaves and adult leaves from the lower, middle, and upper part of the plant. Data presented are means ±SE. Different letters indicate significant differences.

### Hydraulic conductivity and vulnerability to drought-induced embolism

In stem and branch xylem, *k*_s_ varied between 6.58 and 31.14 m^2^ s^–1^ MPa^–1^ ×10^–4^ along the hydraulic pathway. It increased significantly with height, both in the main stem and in branches (Table 2, Fig. 1D). Moreover, *k*_s_ was more than twice as high in the main stem as in branches of the same sampling height. Juvenile branches showed a significantly lower *k*_s_ compared with adult branches at the lowest sampling point (Table 2).

**Table 2.** Xylem hydraulic and anatomical parameters of *Hedera helix*

	Juvenile branches	Adult branches			Main stem	
Parameter	*0–1 m*	*1–2 m*	*7–8 m*	*12–13 m*	*1–2 m*	*7–8 m*
*k*_s_ (m^2^ s^–1^ MPa^–1^ × 10^–4^)	6.58±0.40^a^	9.16±0.75^b^	15.40±0.54^c^	18.67±0.99^d^	24.82±1.96^e^	31.14±1.41^f^
ψ_LC12_ (MPa)	–2.43±0.22^a^	–2.22±0.23^a^	–2.13±0.27^a^	–2.76±0.28^a^	–0.75±0.13^b^	–0.88±0.12^b^
ψ_LC50_ (MPa)	–3.90±0.14^a^	–3.55±0.15^b^	–3.97±0.15^ac^	–4.22±0.14^c^	–2.05±0.11^d^	–2.58±0.11^e^
ψ_LC88_ (MPa)	–5.36±0.15^a^	–4.86±0.14^b^	–5.85±0.13^c^	–5.67±0.12^ac^	–3.32±0.18^d^	–4.32±0.12^e^
*a*	1.38±0.10^ab^	1.58±0.11^a^	1.18±0.11^b^	1.31±0.12^ab^	1.60±0.14^a^	1.19±0.05^b^
*d*_mean_ (µm)	14.60±0.64^a^	15.55±1.27^a^	19.40±1.16^ab^	20.23±2.27^b^	23.12±0.64^bc^	26.63±1.13^c^
*d*_max_ (µm)	35.28	37.79	48.30	56.45	76.72	84.54
*d*_h_ (µm)	23.81±1.05^a^	24.29±1.08^a^	28.17±2.62^a^	30.63±3.58^a^	54.41±1.87^b^	57.19±3.17^b^
(*t*/*b*)^2^	0.023±0.003^a^	0.012±0.001^b^	0.017±0.003^c^	0.020±0.001^d^	0.018±0.002^cd^	0.019±0.001^cd^
area_lumen_ (%)	18.65±1.42^a^	23.97±2.52^a^	30.93±0.45^b^	32.42±2.37^b^	53.51±2.47^c^	56.61±2.65^c^
area_pith_ (%)	56.57±2.09^a^	25.09±1.70^b^	28.23±2.43^b^	30.51±2.63^b^	0.87±0.07^c^	1.20±0.12^c^

Specific hydraulic conductivity (*k*_s_), xylem water potential at 12%, 50%, and 88% loss of xylem conductivity (Ψ_LC12_, Ψ_LC50_, Ψ_LC88_), slope of the vulnerability curve (a), mean conduit diameter (*d*_mean_), maximum conduit diameter (*d*_max_), mean hydraulic diameter (*d*_h_), cell wall thickness to span ratio [(*t*/*b*)^2^], percentage of xylem area covered by conduit lumen (area_lumen_), and percentage of the cross-sectional area occupied by the central pith parenchyma (area_pith_). Data presented are means ±SE. Different letters indicate significant differences.

**Fig. 2.**
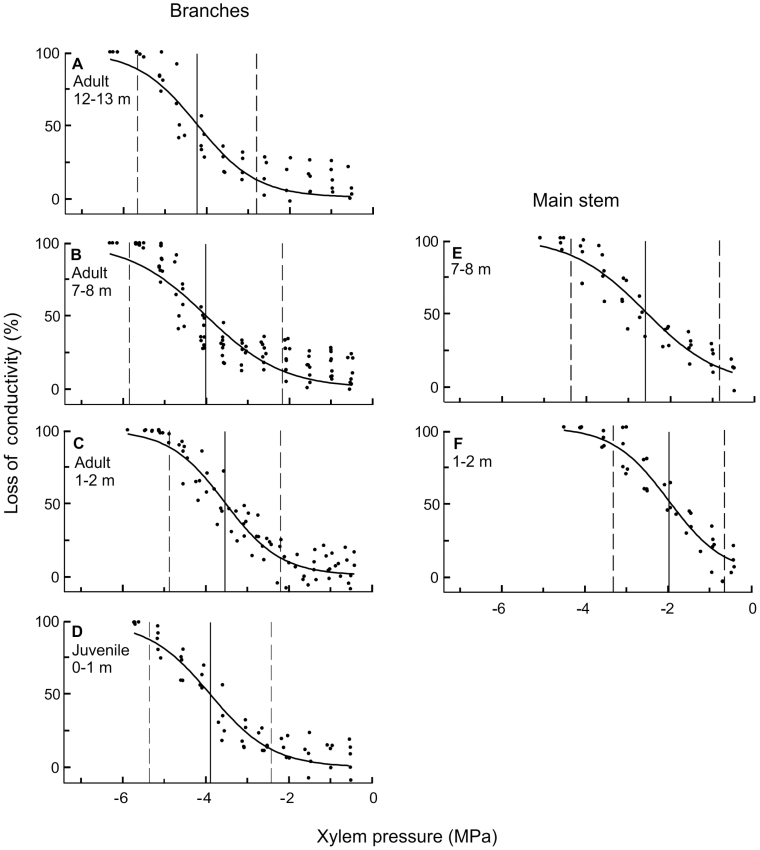
Drought-induced vulnerability (loss of conductivity versus water potential; pooled data of 5–9 curves) of (A–D) branches (adult from the upper, middle, and lower crown, and juvenile) and (E, F) the main stem (middle and lower crown) of *Hedera helix*. Vertical lines on each plot indicate the water potentials at (right to left) 12%, 50%, and 88% loss of conductivity.

Ψ at 50% PLC (Ψ_LC50_) of the main stem varied between –2.05 and –2.58 MPa, and Ψ_LC50_ of branches varied between –3.55 and –4.22 MPa (Table 2). The higher vulnerability of the main stem was also characterized by less negative lower and upper vulnerability thresholds, that is, by significantly less negative Ψ_LC12_ and Ψ_LC88_ compared with respective values for the branches. In adult branches, Ψ_LC50_ decreased with height (Fig. 1E, Fig. 2A–C), and the main stem also showed a more negative Ψ_LC50_ at the higher section compared with the lower section (Table 2). Interestingly, the shift with increasing height was mainly caused by flatter vulnerability curves in both the main stem and branches at higher sampling points (Fig. 2; Table 2). Juvenile branches with a Ψ_LC50_ of –3.90 MPa were slightly more resistant to drought-induced embolism formation than adult branches at 1–2 m height (–3.55 MPa; Table 2).

### Wood characteristics

**Fig. 3.**
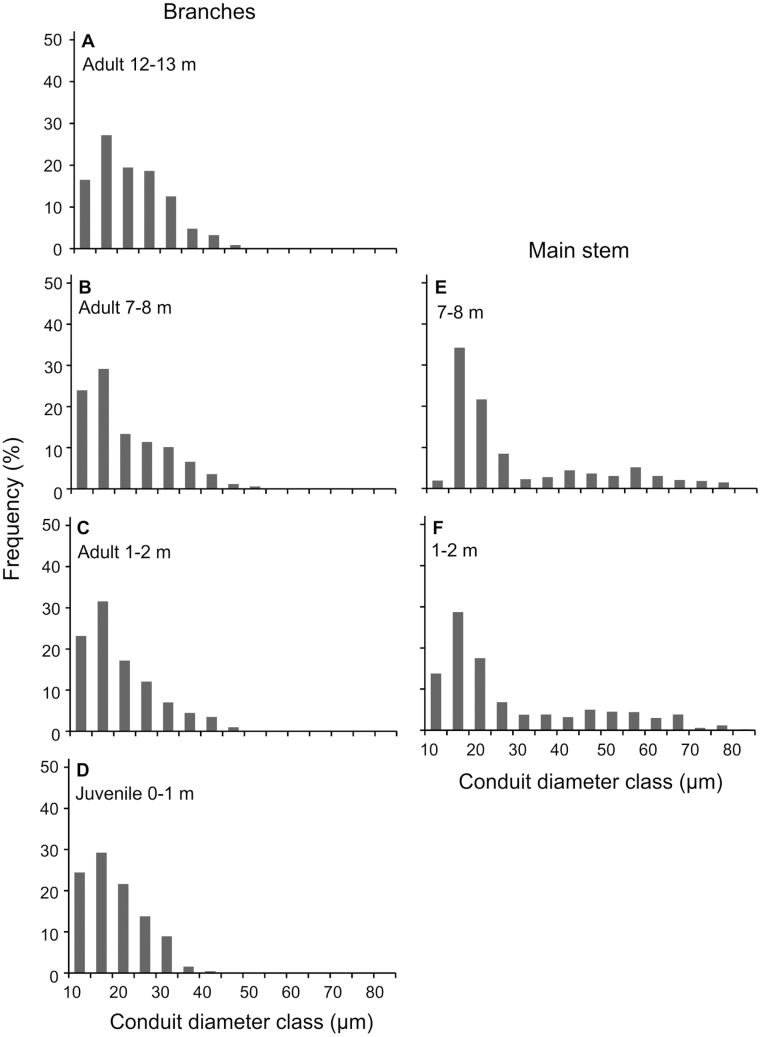
Mean distribution of conduit diameters (5 µm classes) in branches and main stems of *Hedera helix.* Data of five samples per sampling height were pooled.

The main stem of *H. helix* was characterized by wider conduits compared with the branches, with a *d*_mean_ up to 26.63 µm (branches 20.23 µm), *d*_h_ up to 57.19 µm (branches 30.63 µm), and *d*_max_ up to 84.54 µm (branches 56.45 µm; Table 2). Conduit diameters (i.e. *d*_mean_, *d*_h_, and *d*_max_) increased with height in both branches and the main stem (Table 2, Fig. 1F–H) and the frequency distribution showed a shift to wider conduits with increasing height (Fig. 3). The xylem of branches contained a higher proportion of fibres than was found in the stem (see Supplementary Fig. S1 at *JXB* online), resulting in a lower area_lumen_. This applied especially to juvenile branches, with an area_lumen_ of 18.65% (compared with 56.61% in the upper stem; Table 2). Moreover, juvenile branches were characterized by extensive pith parenchyma in the centre and a narrow surrounding xylem area, resulting in a high area_pith_ (56.6%; Table 2), while adult branches of the same diameter exhibited a smaller pith and thus a higher proportion of xylem area.

Cell wall reinforcement was highest in juvenile branches, with a (*t*/*b*)^2^ of 0.023, and lowest in adult branches at the lowest sampling point (0.012). If only adult branches were considered, (*t*/*b*)^2^ increased significantly with height (*R*^2^=0.76, *P*<0.001), but due to the deviance of juvenile branches no overall correlation of branch cell wall reinforcement with height was detected (Fig. 1I). At 1–2 m height, (*t*/*b*)^2^ was significantly higher in the main stem than in adult branches (Table 2).

**Fig. 4.**
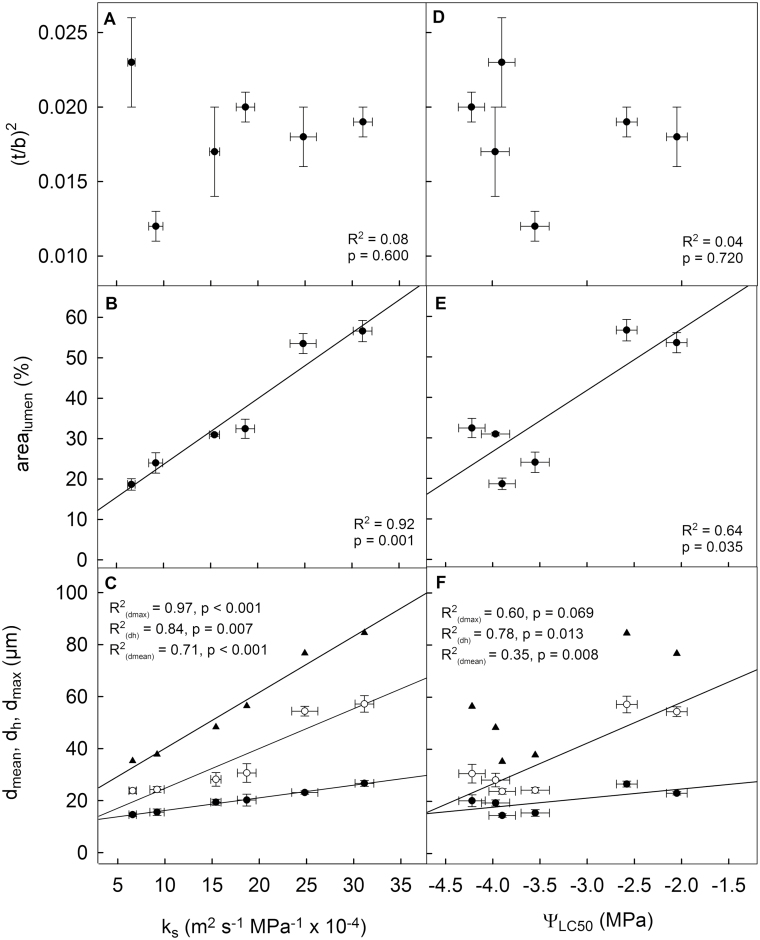
Correlation of wood anatomical parameters with specific hydraulic conductivity (*k*_s_) and vulnerability to cavitation (water potential at 50% loss of conductivity; Ψ_LC50_). (A, D) Cell wall reinforcement [(*t*/*b*)^2^], (B, E) percentage of xylem area covered by conduit lumen (area_lumen_), and (C, F) mean conduit diameter (*d*_mean_; filled circles), mean hydraulic diameter (*d*_h_; open circles), and maximum diameter (*d*_max_; triangles). Data presented are means ±SE of juvenile branches, adult branches, and the main stem from different sampling heights.

Correlation analysis between *k*_s_ and wood anatomical parameters revealed a positive correlation of *k*_s_ with *d*_mean_ (*R*^2^=0.71), *d*_h_ (*R*^2^=0.84), *d*_max_ (*R*^2^=0.97), and area_lumen_ (*R*^2^=0.92), but not with (*t*/*b*)^2^ (*R*^2^=0.08, *P*=0.600; Fig. 4A–C). Ψ_LC50_ was positively correlated with *d*_mean_ (*R*^2^=0.35), *d*_h_ (*R*^2^=0.78), and area_lumen_ (*R*^2^=0.64), but not with *d*_max_ (*R*^2^=0.60, *P*=0.069) or (*t*/*b*)^2^ (*R*^2^=0.04, *P*=0.720; Fig. 4D–F).

### Growth

Tree ring analysis revealed an age of 46–50 years at a minimum for the studied individual ivy plants (see Supplementary Fig. S2); unfortunately, the first rings near the pith could not be well distinguished in all discs. The annual rings of the main stem at 7.5 m height were significantly narrower than those at 1.5 m height for several years, but reached the same width during recent years, with a width of ~0.8 mm per year.

## Discussion

The present study revealed clear differences between the main stem and branches, as well as pronounced vertical gradients in leaf osmotic parameters, hydraulic safety and efficiency, and xylem anatomical features in the liana *H. helix*. Results indicate that the coordination of carbon and water economies with respect to the tall growth form and a vertical light gradient forces non-self-supporting plants to follow within-plant hydraulic adjustments that are similar to those of trees.

### General hydraulic strategy

Efficient and safe water transport from the base to the top is critical for plant growth and survival, especially if large leaf areas in the upper canopy are supported by long and narrow main stems. The present study indicates that ivy follows an efficient but risky hydraulic strategy in terms of a high *k*_s_ in combination with a comparably high Ψ_LC50_ in the main stem. This is consistent with previous research on tropical lianas (e.g. Van der Sande *et al.*, 2013; Chen *et al.*, 2017). The *k*_s_ measured for *H. helix* (up to 18.67 m^2^ s^–1^ MPa^–1^ × 10^–4^ in branches and up to 31.14 m^2^ s^–1^ MPa^–1^ × 10^–4^ in the main stem; Table 2) was higher than mean values reported for temperate angiosperm trees, but lower than *k*_s_ values in vines and most tropical lianas (Gartner *et al.*, 1990; Carvalho *et al.*, 2015; Chen *et al.*, 2017). This is not surprising, as extremely wide vessels (>100 µm), which were found in many tropical lianas and vines (e.g. Gartner *et al.*, 1990; Ewers and Fisher, 1991), were not present in ivy (Table 2, Fig. 3). However, the higher *k*_s_ compared with those of trees can compensate for the smaller xylem cross-sectional area of the liana and enable high flow rates despite the relatively narrow main stem.

The strong down-regulation of stomatal conductance in ivy at vapour pressure deficits greater than 0.5 kPa (Leuzinger *et al.*, 2011) implies that, despite its high *k*_s_ in the main stem, ivy is not maximizing gas exchange during the middle of the day but avoids low Ψ. Accordingly, Ψ values were reported not to fall below –1.3 to –1.7 MPa at midday (at 23 m height). The derived assumption of Leuzinger *et al.* (2011) that this strategy originates from a high stem vulnerability to the formation of embolism can be confirmed by findings of the present study, as Ψ_LC50_ was between –2.05 and –2.58 MPa in the main stem (Table 2). At approximately –1.5 MPa, a PLC of 20–30% can be expected in the main stem (Fig. 2E–F), which forces ivy to close stomata before more critical water potentials in the stem are reached. The vulnerability of the main stem of ivy is probably higher than of typical host trees such as *Larix decidua*, *Picea abies*, *Quercus petraea*, and *Carpinus betulus.* Unfortunately, available vulnerability data for these species (Ψ_LC50_ between –3.50 and –3.98 MPa; Choat *et al.*, 2012) and most other tree species are based on branch samples, while information on the vulnerability of main stems is scarce, due to methodological difficulties. Better knowledge of the hydraulics of mature plant trunks would be a prerequisite (see also Johnson *et al.*, 2016) to better understand the hydraulic architecture of trees as well as lianas. Lower embolism resistance of lianas compared with trees has also been reported for tropical species and was based on comparisons of branches or seedling stems (Van der Sande *et al.*, 2013; Chen *et al.*, 2017). The investigated tropical lianas showed an overall higher vulnerability than that of ivy (Ψ_LC50_ between –0.29 and –1.57 MPa), while vine-like bamboos adapted to drier habitats can reach Ψ_LC50_ values below –4 MPa (Cochard *et al.*, 1994). Further studies on temperate lianas would be needed to elucidate general trends in the variation in hydraulic efficiency and safety across climatic zones and precipitation gradients in climbing plants.

### Hydraulics of the main stem versus branches

The water transport system in the main stem of ivy was distinctly more efficient than in branches, with a *k*_s_ twice as high (Table 2). In contrast, the main stem was less embolism resistant, as its Ψ_LC50_ was up to 1.5 MPa less negative compared with branches from the same height (Table 2). The higher hydraulic efficiency and lower safety in the main stem compared with branches corresponds to patterns reported for lianas from semi-arid regions (Carvalho *et al.*, 2015) and several angiosperm and gymnosperm trees (Zimmermann, 1978; Domec *et al.*, 2006; Beikircher and Mayr, 2008; Johnson *et al.*, 2016). Due to the small cross-sectional area of the main stem, high *k*_s_ is apparently essential for temperate lianas to prevent a strong drop of Ψ during transpiration, and limited embolism resistance is probably the consequence of a trade-off between safety and efficiency (i.e. based on pit characteristics and conduit size, grouping, and connection; Wheeler *et al.*, 2005). Furthermore, comparably low hydraulic safety in the main stem might be sufficient for lianas growing under moist conditions, and even more so if they have conservative stomatal regulation (see above), as is the case in ivy. However, it remains unclear how temperate lianas exposed to frequent drought stress may balance hydraulic efficiency and safety.

The higher hydraulic safety observed in branches compared with the main stem in ivy can be related to the branches’ position at the distal end of the water transport pathway, where lower Ψ values occur during transpiration; a similar situation occurs in trees. Furthermore, branches of ivy showed a higher proportion of supporting tissue and smaller conduits in the xylem compared with the main stem (Supplementary Fig. S1), which may not only lead to lower *k*_s_ but also correspond to lower Ψ_LC50_ (Table 2). Rigid xylem structures in the branches are probably formed for mechanical reasons, which is especially relevant in adult crown parts of ivy, where shoots do not form adhesive rootlets (Bauer and Bauer, 1980) and thus cannot statically rely on the host tree.

### Height-related hydraulic changes

With increasing height, Ψ decreases because of gravity and cumulative resistance in the hydraulic pathway (Tyree and Ewers, 1991). Distal parts of the plant thus require adaptations to maintain a favourable water status at lower Ψ. Accordingly, in *H. helix*, the Ψ_o_, Ψ_tlp_, and ε of leaves, as well as the Ψ_LC50_ of stem and branches, decreased with increasing height (Fig. 1, Table 2), to counter potential problems such as wilting or embolism formation. Furthermore, *k*_s_ increased in branches with increasing insertion height, and between 1 m and 7 m along the main stem (Fig. 1D, Table 2). Higher *k*_s_ in upper branches has also been reported for several tall tree species (Clearwater and Meinzer, 2001; Burgess *et al.*, 2006; Domec *et al.*, 2006; Yoshimura 2011), indicating that hydraulic traits are coordinated in a similar way within both self-supporting and non-self-supporting tall plants to ensure water supply to a large number of leaves with high transpirational demands in the upper crown (Pfautsch *et al.*, 2018) and to compensate for their height ‘disadvantage’ (Zimmermann, 1978; Bettiati *et al.*, 2012). Higher xylem conductivity was the result of larger conduits as well as a higher area_lumen_ and may be crucial to balance hydraulic resistances in the crown (see the section on xylem structure, below, for a detailed discussion). There was no trade-off between safety and efficiency in branches across different heights in ivy, as branches from the upper crown regions showed high hydraulic safety and efficiency.

### Hydraulics of juvenile versus adult branches

Juvenile leaves of *H. helix* did not differ in Ψ_o_ and Ψ_tlp_ compared with adult leaves near the ground (Table 1). Although this may seem unexpected, because juvenile leaves are characterized as shade leaves and adult leaves as sun leaves (Bauer and Bauer, 1980), it has to be borne in mind that those leaves grew under similar low-light conditions. We chose large ivy plants, in which the lowest adult crown parts were situated in the shadow of the upper (host and ivy) crown. The differences between the two life stages were more pronounced with respect to adult leaves at higher levels, where the liana reached the canopy of the host tree, where there are higher light levels and consequently higher photosynthetic and transpiration rates (Leuzinger *et al.*, 2011). Adult leaves from the upper crown showed a better osmotic adaptation to low water potentials (Ψ_o_ –2.05 MPa and Ψ_tlp_ –2.31 MPa; see also Burghardt and Riederer, 2003 for comparison). Cell walls were more elastic in adult than in juvenile leaves, which corresponds to the fact that juvenile leaves are more rigid and morphologically different from adult leaves (Bauer and Bauer, 1980). Moreover, the juvenile and adult life phases differed in *k*_s_ and Ψ_LC50_, with juvenile branches showing a lower xylem efficiency but higher embolism resistance (Table 2). Low *k*_s_ is probably sufficient in juvenile branches because the cumulative area and transpiration rates of juvenile leaves are lower than in adult leaves (Miller and Goodin, 1976, Bauer and Bauer, 1980). In this case, high embolism resistance (Fig. 2D, Table 2) is advantageous as it guarantees permanent, although small, transport capacity. As soon as the plant starts to form a large crown with adult branches, probably in combination with an extended root system, transport capacity may become more important and hydraulic traits characteristic for tall plants more pronounced.

### Xylem structure

Tree ring analyses revealed increasing growth rates during the first 25 years of the life span of the studied ivy plants (Supplementary Fig. S2), when the liana was progressively extending its upper canopy. Mean annual xylem growth during the most recent 5 years settled at 0.82±0.06 mm, comparable to values reported for ivy in alluvial environments by Castagneri *et al.* (2013) (1.05±0.54 mm) and Heuzé *et al.* (2009) (0.77–1.89 mm).

Xylem anatomical parameters differed significantly between the main stem and branches, across heights, and between juvenile and adult branches of *H. helix* (Table 2, Fig. 1F–J) and explained most of the variation in hydraulic efficiency and partly also the variation in hydraulic safety. In accordance with the Hagen–Poiseuille equation, *k*_s_ was highly correlated with *d*_h_, but also with *d*_mean_, *d*_max_, and area_lumen_ (Fig. 4B, C). For several lianas and vines, studies revealed a stronger linkage between hydraulic conductance and conduit size than in trees (Ewers *et al.*, 1989; Gartner *et al.*, 1990). This can be explained by the long vessels and thus comparably minor pit effects (Tyree and Zimmermann, 2002), and by the reduced mechanical function of the wood in non-self-supporting plants (Tyree and Ewers, 1991). Internal xylem anatomical adjustments are crucial for tall plants, as gravitational pressure and path length resistances increase with plant size. Scaling conduit dimensions along the vertical axis can minimize the height effect, maximize the hydraulic conductance for a given carbon investment, and improve equal water supply to all parts of the crown (Mencuccini *et al.*, 2007; Bettiati *et al.*, 2012). Accordingly, conduit widening was empirically observed for trees in several studies (e.g. Anfodillo *et al.*, 2006; Olson *et al.*, 2014; Prendin *et al.*, 2018); it was also reported for non-self-supporting plants by comparing species with varying stem length (interspecific tapering; Olson *et al.*, 2014), but had not previously been analysed within a liana. Although the present study does not allow us to draw detailed conclusions about the axial widening within ivy because of the limited number of sampling points along the stem, it seems likely that this liana behaves like self-supporting trees. According to conduit widening allometry, the diameter varies sharply near the apex but very little near the stem base (Anfodillo *et al.*, 2006), and the small increase in conduit diameter at the trunk base found in ivy (Table 2) is also a frequently observed phenomenon in trees (Bettiati *et al.*, 2012; Pfautsch *et al.*, 2018). The clear increase of conduit dimensions with branch insertion height (Fig. 1F–H) was similarly found in several tree species (Clearwater and Meinzer, 2001; Burgess *et al.*, 2006; Bettiati *et al.*, 2012) and can equalize the hydraulic resistance of all root-to-leaf pathways within the crown. As the water transport distance from the trunk base is longer in distal leaves, they would be hydraulically disadvantaged compared with leaves on lower branches unless larger conduits reduced the hydraulic resistance.

Interrelations between embolism resistance and xylem anatomy were not pronounced in ivy, as Ψ_LC50_ was correlated with *d*_h_, *d*_mean_, and area_lumen_, but not with *d*_max_ and (*t*/*b*)^2^ (Fig. 4D–F), although the higher vulnerability of adult versus juvenile branches, and of lower versus upper branches, was associated with lower (*t*/*b*)^2^ (Table 2). A relationship between embolism resistance and (*t*/*b*)^2^ could be expected, as lower Ψ_LC50_ values are commonly associated with higher wall reinforcement to reduce wall bending and the risk of conduit collapse under negative pressure (Hacke and Sperry, 2001; Wheeler *et al.*, 2005). In contrast, the relationship with *d*_h_ might be indirect via pit properties (the ‘pit area hypothesis’). For instance, microtomographic observations on *Laurus nobilis* showed that at moderate water stress the fraction of embolized vessels was highest in the upper vessel diameter classes (Nardini *et al.*, 2017). Vulnerability to freeze–thaw-induced embolism, which is more directly linked to conduit diameter, was not considered in our study. Nevertheless, this aspect has been suggested to be relevant for temperate lianas (Ewers, 1985; Tibbetts and Ewers, 2000) and should be investigated in further studies.

Overall, the close linkage between wood anatomical traits and *k*_s_ indicates a strong selective pressure for hydraulic efficiency in temperate lianas. Balance of hydraulic efficiency with hydraulic safety is achieved in *H. helix*, as in other liana species (Carlquist, 1985), by a characteristic combination of wide and efficient vessels with narrow and probably embolism-resistant vessels in the main stem (Fig. 3).

## Conclusions

The temperate liana *H. helix* shows a hydraulic architecture adapted to its climbing habit, tall growth form, and occurrence in rather moist forests, although its hydraulic characteristics are less pronounced than in better-studied tropical lianas. Anatomical features of xylem compensate for the limited cross-sectional area of the main stem, ensuring high hydraulic efficiency and water supply of a large leaf area. Low transport resistances, together with the reported sensitive stomatal response, can prevent a strong drop in Ψ upon transpiration and thus relatively high Ψ_LC50_ in the stem may not be critical for performance and survival.

Within-plant hydraulic variations were pronounced between branches and the main stem and with increasing branch insertion height, but small between the juvenile and adult life phases. Patterns of xylem anatomy and hydraulic efficiency and safety are in accordance with the pattern reported for trees, suggesting that lianas apply the same effective architectural design as self-supporting plants to reduce the path length effect on the total hydraulic resistance and guarantee efficient and safe water supply to all parts of the crown. This study highlights the necessity of studying whole-plant hydraulic architecture, including varying growth forms, to better understand the hydraulics of tall plants and underlying functional trade-offs.

## Author contributions

SM and AG led the study; all authors contributed to the experimental design and methodical developments, conducted field and laboratory measurements, and performed data analysis; AG prepared the article with contributions from all the authors; SM supervised and complemented the writing.

## Supplementary data

Supplementary data are available at *JXB* online.

**Fig. S1.** Cross-sections of a *Hedera helix* branch and main stem.

**Fig. S2.** Growth (annual ring width) of *Hedera helix* stems from the years 1965 to 2015.

Supplementary Figures S1 and S2
