# Supplementary material for: Are hydraulic patterns of lianas different from trees? New insights from Hedera helix
Source: J Exp Bot. 2019 Feb 22;70(10):2811–22. doi: 10.1093/jxb/erz071 (PMC6506770; doi:10.1093/jxb/erz071)
Supplement: Supplementary Figures S1 and S2 [file erz071_suppl_supplementary-figures-s1-s2.pdf]

Supplemental Material

Are hydraulic patterns of lianas different from trees? New insights from *Hedera helix*

Ganthaler A., Marx K., Beikircher B., Mayr S.

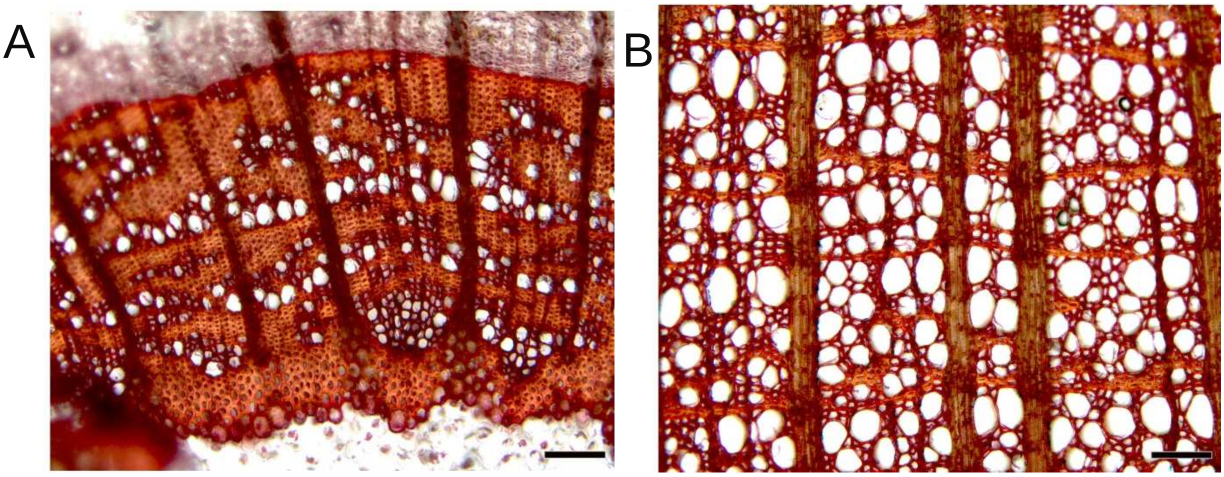

Suppl. Figure 1. Cross sections of an adult branch (A) and the main stem (B) of *Hedera helix* at 7-8 m height. Staining with Etzold solution (fuchsin/safranine/astrablue), Bars = 100 µm.

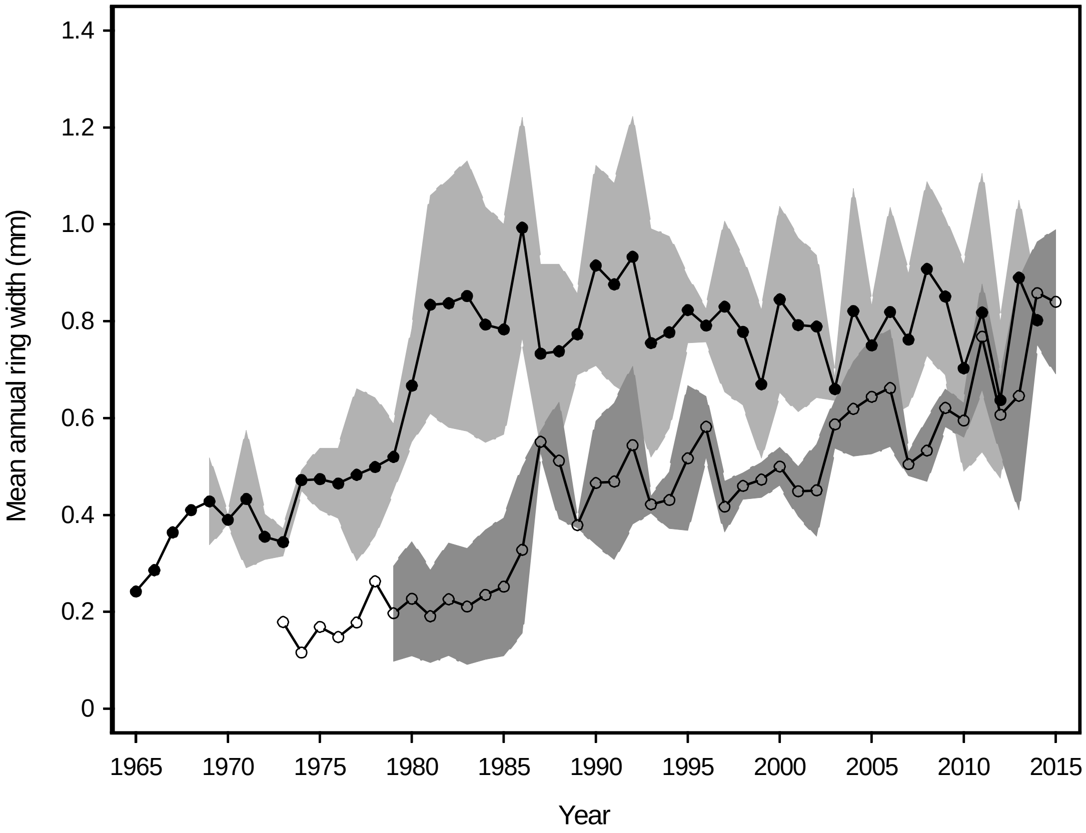

Suppl. Figure 2. Mean annual ring width (symbols) and SE (grey areas; n = 3) of *Hedera helix* main stems at 1.5 m (solid circles, light grey area) and 7.5 m height (open circles, dark grey area) for the years 1965 to 2015. Symbols outside the grey areas show single values of the oldest individual.
